# Genetic diversity and structure of tea plant in Qinba area in China by three types of molecular markers

**DOI:** 10.1186/s41065-018-0058-4

**Published:** 2018-05-09

**Authors:** Yu Zhang, Xiaojuan Zhang, Xi Chen, Wang Sun, Jiao Li

**Affiliations:** 1 School of Biological Science and Engineering, Shaanxi University of Technology, Hanzhong, Shaanxi Province 723000 People’s Republic of China; 2 Qili Food and Drug Supervision and Management Institute, Hanzhong, Shaanxi Province 723000 People’s Republic of China; 3 Hanzhong Institute of Agricultural Sciences, Hanzhong, Shaanxi Province 723000 People’s Republic of China

**Keywords:** *Camellia sinensis*, Marker efficiency, Correlation coefficient, Genetic diversity, Population structure

## Abstract

**Background:**

Qinba area has a long history of tea planting and is a northernmost region in China where *Camellia sinensis* L. is grown. In order to provide basic data for selection and optimization of molecular markers of tea plants. 118 markers, including 40 EST-SSR, 40 SRAP and 38 SCoT markers were used to evaluate the genetic diversity of 50 tea plant (*Camellia sinensis.*) samples collected from Qinb. tea germplasm, assess population structure.

**Results:**

In this study, a total of 414 alleles were obtained using 38 pairs of SCoT primers, with an average of 10.89 alleles per primer. The percentage of polymorphic bands (PPB), polymorphism information content (PIC), resolving power (Rp), effective multiplex ratio (EMR), average band informativeness (Ib_av_), and marker index (MI) were 96.14%, 0.79, 6.71, 10.47, 0.58, and 6.07 respectively. 338 alleles were amplified via 40 pairs of SRAP (8.45 per primer), with PPB, PIC, Rp, EMR, Ib_av,_ and MI values of 89.35%, 0.77, 5.11, 7.55, 0.61, and 4.61, respectively. Furthermore, 320 alleles have been detected using 40 EST-SSR primers (8.00 per primer), with PPB, PIC, Rp, EMR, Ib_av_, and MI values of 94.06%, 0.85, 4.48, 7.53, 0.56, and 4.22 respectively. These results indicated that SCoT markers had higher efficiency.

Mantel test was used to analyze the genetic distance matrix generated by EST-SSRs, SRAPs and SCoTs. The results showed that the correlation between the genetic distance matrix based on EST-SSR and that based on SRAP was very small (*r* = 0.01), followed by SCoT and SRAP (*r* = 0.17), then by SCoT and EST-SSR (*r* = 0.19).

The 50 tea samples were divided into two sub-populations using STRUCTURE, Neighbor-joining (NJ) method and principal component analyses (PCA). The results produced by STRUCTURE were completely consistent with the PCA analysis. Furthermore, there is no obvious relationship between the results produced using sub-populational and geographical data.

**Conclusion:**

Among the three types of markers, SCoT markers has many advantages in terms of NPB, PPB, Rp, EMR, and MI. Nevertheless, the values of PIC showed different trends, with the highest values generated with EST-SSR, followed by SCoT and SRAP. The average band informativeness showed similar trends. Correlation between genetic distances produced by three different molecular markers were very small, thus it is not recommended to use a single marker to evaluate genetic diversity and population structure. It is hence suggested that combining of different types of molecular markers should be used to evaluate the genetic diversity and population structure. It also seems crucial to screen out, for each type of molecular markers, core markers of *Camellia sinensis*. This study revealed that genes of exotic plant varieties have been constantly integrated into the gene pool of Qinba area tea. A low level of genetic diversity was observed; this is shown by an average coefficient of genetic similarity of 0.74.

## Background

Evaluation of genetic diversity and population structure has significant implications for genetic improvement in plant breeding. It has been well established that the genetic basis of biological organisms is concealed within the genome sequence, and that base-pair substitution, insertion, deletion, and other alterations can lead to genetic diversity; the diversity of organisms are manifested through phenotypic, chromosomal and proteomic differences. DNA molecular markers, having stable performance, high polymorphism and other properties, are increasingly employed in taxonomical, genetic evolutionary, breeding, and cloning studies. The use of different molecular markers and different primers for a same marker may result in amplification of distinct regions of the genome. Theoretically, higher numbers of polymorphic markers used are associated with wider amplified regions that covers the entire genome and more accurate results.

EST-SSR (Expressed Sequence Tag-Simple Sequence Repeat) molecular markers have been widely used with many species and for many applications, such as genetic linkage mapping, comparative mapping, and evaluation of genetic diversity [1–5]. SRAP (Sequence related amplified polymorphism) was first used on *Brassica* in 2001 by Li G [6]. The genetic diversity and population structure analysis of *Camellia sinensis* by SRAP [7–12] have already been reported. SCoT (Start codon targeted polymorphism) marker was designed according to the Kozak sequence pattern and was developed after the discovery of the conservativeness of the initiation codon ATG (+ 1, + 2, + 3) flanking sequences, in which the positions + 4, + 7, + 8, and + 9 are occupied by nucleotides G, A, C, and C, respectively. These seven nucleotides are generally conserved. At positions − 3, − 6, and − 9, G is the usual nucleotide. Primers can therefore be designed according to the conservativeness of the initiation sequence SCoT marker allows single primer amplification of the region between two genes. Bertrand et al. first applied this marker on *Oryza sativa* [13]. Lately, SCoT molecular marker has been used to access the genetic diversity of plant species such as *Saccharum spontaneum L* [14], *Dactylis glomerata* [15], *Mangifera indica* [16], *Arachis hypogaea* [17], *Saccharum officinarum* [18], *Podocarpus macrophyllus* [19] and *Paeonia suffruticosa* [20]. Nevertheless, no similar study has been conducted on *Camellia sinensis*. Tea plant is an allogamous species; theoretically, after prolonged spontaneous hybridization, the genetic background of tea plant should be increasingly complex.

China is one of the main sources of tea germplasms. Currently, there are 1,100,000 ha of tea planting area, with different regions growing different types and different varieties of tea according to topographic, soil, and climatic characteristics. Xinan, Huanan, Jiangnan, and Jiangbei represent the four main districts of tea planting area in China. The Qinba area belongs to the Jiangbei district. In this research, 50 tea varieties, including those collected from different districts, common tea plant species, as well as local species in the Qinba area, were genotyped with EST-SSR, SRAP, and SCoT markers. Herein we constructed three types of molecular marker dataset which have important applications in diversity analysis, marker efficiency analysis, and correlation analysis that use these marker systems. Our study allowed the establishment of population structure, providing significant insights into the selection of molecular markers for tea plant breeding.

## Results and discussion

### Marker efficiency analysis

In this study, three types of molecular markers were used to differentiate tea plant accessions. A total of 1072 bands were produced using 118 primer pairs. 38 SCoT, 40 SRAP and 40 EST-SSR primers were selected for further studies according to the percentage of polymorphic bands (PPB), polymorphism information content (PIC) and the degree of clear band selected markers using six selected genotypes (Table 1). A total of 414, 338, and 320 bands were obtained using SCoT, SRAP and EST-SSR markers, respectively from the 50 test materials, which included 398, 302, and 301 polymorphic bands, with PPBs of 96.13%, 89.35%, and 94.06%. Comparisons of the three types of markers are shown in Table 2. SCoT markers have a higher marker efficiency and are excellent for the appraisal of polymorphic loci, except that its polymorphic information content is lower than that of EST-SSR.

**Table 1:** Amplification results of EST-SSR, SRAP, and SCoT primers

Primer name	Sequence(5′-3′)	Total number of bands(TNB)	The number of polymorphic bands(NPB)	Percentage of polymorphic bands(PPB)%	Polymorphism information content(PIC)
SCoT1	CAACAATGGCTACCACCC	6	5	83.33	0.98
SCoT2	CAACAATGGCTACCACCG	10	10	100	0.98
SCoT3	CAACAATGGCTACCACCT	7	7	100	0.98
SCoT4	CAACAATGGCTACCACGA	11	11	100	0.84
SCoT5	CAACAATGGCTACCACGC	13	13	100	0.96
SCoT6	CAACAATGGCTACCACGT	10	10	100	0.89
SCoT7	CAACAATGGCTACCAGCA	13	12	92.31	0.88
SCoT8	CAACAATGGCTACCAGCC	12	11	91.67	0.95
SCoT9	ACGACATGGCGACCATCG	10	10	100	0.92
SCoT10	ACGACATGGCGACCACGC	8	8	100	0.93
SCoT11	ACCATGGCTACCACCGAC	9	9	100	0.87
SCoT12	ACCATGGCTACCACCGCC	13	12	92.31	0.90
SCoT13	ACCATGGCTACCACCGCG	12	10	83.33	0.87
SCoT14	ACGACATGGCGACCCACA	13	12	92.31	0.91
SCoT15	ACCATGGCTACCACCGGG	14	14	100	0.98
SCoT16	CCATGGCTACCACCGCCA	10	9	90	0.91
SCoT17	CCATGGCTACCACCGCAC	10	10	100	0.96
SCoT18	CCATGGCTACCACCGCAG	6	6	100	0.94
SCoT19	ACCATGGCTACCACCGCA	12	12	100	0.87
SCoT20	CATGGCTACCACCGGCCC	12	12	100	0.95
SCoT21	ACGACATGGCGACCAACT	9	9	100	0.93
SCoT22	ACGACATGGCGACCAACC	11	11	100	0.95
SCoT23	ACGACATGGCGACCATCC	6	6	100	0.77
SCoT24	ACGACATGGCGACCACGG	10	10	100	0.95
SCoT25	ACGACATGGCGACCACGT	13	12	92.31	0.89
SCoT26	ACCATGGCTACCACCGCT	10	10	100	0.97
SCoT27	ACCATGGCTACCACCGGA	9	9	100	0.90
SCoT28	CCATGGCTACCACCGCCC	13	13	100	0.77
SCoT29	CCATGGCTACCACCGCCG	13	11	100	0.89
SCoT30	CCATGGCTACCACCGGCT	4	4	100	0.92
SCoT31	CCATGGCTACCACCGCAA	10	10	100	0.89
SCoT32	CCATGGCTACCACCGCAT	7	7	100	0.98
SCoT33	CATGGCTACCACCGGCCA	17	17	100	0.87
SCoT34	CCATGGCGACCACCGGCA	11	10	90.91	0.71
SCoT35	CCATGGCGACCACCGGCG	17	15	88.24	0.81
SCoT36	CCATGGCGACCACCGGCC	14	13	92.86	0.68
SCoT37	CCATGGCGACCACCGCCG	16	16	100	0.74
SCoT38	CCATGGTCACCACCGGCG	13	12	92.31	0.56
SRAP1	Me1:TGAGTCCAAACCGGATA	8	8	100	0.79
	Em6: GACTGCGTACGAATTCTA				
SRAP2	Me1:TGAGTCCAAACCGGATA	7	7	100	0.87
	Em10:GACTGCGTACGAATTCCA				
SRAP3	Me1:TGAGTCCAAACCGGATA	8	8	100	0.82
	Em13:GACTGCGTACGAATTTGA				
SRAP4	Me2:TGAGTCCAAACCGGCTT	9	8	88.89	0.98
	Em5:GACTGCGTACGAATTCTG				
SRAP5	Me2:TGAGTCCAAACCGGCTT	8	7	87.5	0.94
	Em9:GACTGCGTACGAATTCAT				
SRAP6	Me2:TGAGTCCAAACCGGCTT	9	8	88.89	0.95
	Em11:GACTGCGTACGAATTCAC				
SRAP7	Me2:TGAGTCCAAACCGGCTT	10	8	80	0.85
	Em14:GACTGCGTACGAATTCTT				
SRAP8	Me3:TGAGTCCAAACCGGCTG	8	8	100	0.86
	Em1:GACTGCGTACGAATTATC				
SRAP9	Me3:TGAGTCCAAACCGGCTG	9	9	100	0.78
	Em8:GACTGCGTACGAATTTCC				
SRAP10	Me4:TGAGTCCAAACCGGCCA	12	10	83.33	0.95
	Em1:GACTGCGTACGAATTATC				
SRAP11	Me4:TGAGTCCAAACCGGCCA	11	10	90.9	0.98
	Em2:GACTGCGTACGAATTTAT				
SRAP12	Me4:TGAGTCCAAACCGGCCA	7	7	100	0.99
	Em3:GACTGCGTACGAATTTAG				
SRAP13	Me4:TGAGTCCAAACCGGCCA	8	8	100	0.96
	Em7:GACTGCGTACGAATTTCT				
SRAP14	Me5:TGAGTCCAAACCGGGTA	10	10	100	0.96
	Em2:GACTGCGTACGAATTTAT				
SRAP15	Me5:TGAGTCCAAACCGGGTA	10	9	90	0.93
	Em3:GACTGCGTACGAATTTAG				
SRAP16	Me5:TGAGTCCAAACCGGGTA	9	9	100	0.98
	Em5:GACTGCGTACGAATTCTG				
SRAP17	Me6:TGAGTCCAAACCGGTGA	11	10	90.9	0.92
	Em2:GACTGCGTACGAATTTAT				
SRAP18	Me6:TGAGTCCAAACCGGTGA	12	9	81.82	0.85
	Em3:GACTGCGTACGAATTTAG				
SRAP19	Me7:TGAGTCCAAACCGGTGT	13	11	84.62	0.95
	Em4r:GACTGCGTACGAATTTGT				
SRAP20	Me7:TGAGTCCAAACCGGTGT	12	11	91.67	0.84
	Em9:GACTGCGTACGAATTCAT				
SRAP21	Me7:TGAGTCCAAACCGGTGT	7	6	75	0.86
	Em12:GACTGCGTACGAATTCAA				
SRAP22	Me7:TGAGTCCAAACCGGTGT	7	7	100	0.92
	Em14:GACTGCGTACGAATTCTT				
SRAP23	Me8:TGAGTCCAAACCGGACC	5	5	100	0.96
	Em2:GACTGCGTACGAATTTAT				
SRAP24	Me8:TGAGTCCAAACCGGACC	11	8	72.73	0.94
	Em4:GACTGCGTACGAATTTGT				
SRAP25	Me8:TGAGTCCAAACCGGACC	10	8	80	0.95
	Em8:GACTGCGTACGAATTTCC				
SRAP26	Me8:TGAGTCCAAACCGGACC	8	7	87.5	0.94
	Em13:GACTGCGTACGAATTTGA				
SRAP27	Me9:CTTACTTAGACCGGAGT	7	6	85.71	0.97
	Em2:GACTGCGTACGAATTTAT				
SRAP28	Me9:CTTACTTAGACCGGAGT	9	7	77.78	0.97
	Em3:GACTGCGTACGAATTTAG				
SRAP29	Me9:CTTACTTAGACCGGAGT	5	5	100	0.98
	Em5:GACTGCGTACGAATTCTG				
SRAP30	Me9:CTTACTTAGACCGGAGT	6	6	100	0.99
	Em6:GACTGCGTACGAATTCTA				
SRAP31	Me9:CTTACTTAGACCGGAGT	7	6	85.71	1.00
	Em9:GACTGCGTACGAATTCAT				
SRAP32	Me9:CTTACTTAGACCGGAGT	8	7	87.5	0.90
	Em14:GACTGCGTACGAATTCTT				
SRAP33	Me10:TGAGTCCAAACCGGAAA	9	7	77.78	0.97
	Em5:GACTGCGTACGAATTCTG				
SRAP34	Me10:TGAGTCCAAACCGGAAA	8	8	100	0.96
	Em7:GACTGCGTACGAATTTCT				
SRAP35	Me10:TGAGTCCAAACCGGAAA	5	5	100	0.93
	Em13:GACTGCGTACGAATTTGA				
SRAP36	Me10:TGAGTCCAAACCGGAAA	8	6	75	0.99
	Em14:GACTGCGTACGAATTCTT				
SRAP37	Me11:GTACATAGAACCGGAGT	6	5	83.33	0.96
	Em4:GACTGCGTACGAATTTGT				
SRAP38	Me11:GTACATAGAACCGGAGT	6	5	83.33	1.00
	Em5:GACTGCGTACGAATTCTG				
SRAP39	Me11:GTACATAGAACCGGAGT	5	5	100	0.97
	Em7:GACTGCGTACGAATTTCT				
SRAP40	Me11:GTACATAGAACCGGAGT	10	8	80	0.98
	Em14:GACTGCGTACGAATTCTT				
EST-SSR2	F:GTCAAGAAAGCTCAAGGC	11	10	90.91	0.96
	R:GATGGGCTTGTCTTCGTC				
EST-SSR4	F:GTCAAGAAAGCTCAAGGC	7	7	100	0.96
	R:TGTCTTGTGACCAAATTGAC				
EST-SSR5	F:GTCAAGAAAGCTCAAGGC	6	6	100	0.89
	R:TGAAGTGGCGGCGGAAGA				
EST-SSR7	F:GTCAAGAAAGCTCAAGGC	5	5	100	0.97
	R:GTCAAGTCAAAAACGCCG				
EST-SSR9	F:CCACCGTTGATTCTACTTT	12	11	91.67	0.97
	R:AACAGAGCATACCCAGAAG				
EST-SSR14	F:CCACCGTTGATTCTACTTT	9	9	100	0.99
	R:AAGACCCATACAAAAGATACT				
EST-SSR15	F:CCACCGTTGATTCTACTTT	8	7	87.5	0.92
	R:GATGGGCTTGTCTTCGTC				
EST-SSR17	F:CCACCGTTGATTCTACTTT	9	9	100	0.89
	R:GTCAAGTCAAAAACGCCG				
EST-SSR19	F:CCACCGTTGATTCTACTTT	6	6	100	0.98
	R:CTGCGAACCCTCTTGACC				
EST-SSR20	F:ATCCACCGTATGATGCT	5	5	100	0.98
	R:GATGGGCTTGTCTTCGTC				
EST-SSR23	F:GAATCAGTGAATAAAGCGTGTA	8	8	100	0.97
	R:TGAAGTGGCGGCGGAAGA				
EST-SSR24	F:GAATCAGTGAATAAAGCGTGTA	7	7	100	0.99
	R:TTGGTAGCCTCTTCTTTTG				
EST-SSR26	F:CTCCGATTACTTTCTTCC	7	6	85.71	0.98
	R:GATGACGATGGAGTGGG				
EST-SSR27	F:CATAGTAGAGAAGACCACCA	8	7	87.5	0.99
	R:GATGGGCTTGTCTTCGTC				
EST-SSR30	F:CATAGTAGAGAAGACCACCA	7	6	85.71	0.94
	R:GATGACGATGGAGTGGG				
EST-SSR33	F:GAAAGTGCGAAACCAAAC	3	3	100	0.98
	R:TGAAGTGGCGGCGGAAGA				
EST-SSR35	F:GAAAGTGCGAAACCAAAC	6	6	100	0.93
	R:GTCAAGTCAAAAACGCCG				
EST-SSR38	F:GAAAGTGCGAAACCAAAC	9	9	100	0.94
	R:CTGCGAACCCTCTTGACC				
EST-SSR39	F:CAAGCAATACATACACACA	16	14	87.5	0.86
	R:AAGACCCATACAAAAGATACT				
EST-SSR43	F:CAAGCAATACATACACACA	5	5	100	0.98
	R:AAAACAAGCCACCTCTA				
EST-SSR47	F:CTCTTGATTGGTGCCTTTA	11	10	90.91	0.92
	R:AAGACCCATACAAAAGATACT				
EST-SSR49	F:CTCTTGATTGGTGCCTTTA	9	9	100	0.92
	R:GATGGGCTTGTCTTCGTC				
EST-SSR50	F:CTCTTGATTGGTGCCTTTA	7	7	100	0.98
	R:TGTCTTGTGACCAAATTGAC				
EST-SSR52	F:CTCTTGATTGGTGCCTTTA	12	11	91.67	0.82
	R:TGAAGTGGCGGCGGAAGA				
EST-SSR53	F:CATTGCCTTGATGCTGA	9	9	100	0.90
	R:AAGACCCATACAAAAGATACT				
EST-SSR56	F:CATTGCCTTGATGCTGA	9	9	100	0.91
	R:GATGGGCTTGTCTTCGTC				
EST-SSR58	F:CATTGCCTTGATGCTGA	12	10	83.33	0.98
	R:TGAAGTGGCGGCGGAAGA				
EST-SSR59	F:CATTGCCTTGATGCTGA	13	11	84.62	0.96
	R:GTCAAGTCAAAAACGCCG				
EST-SSR64	F:CCACCGTTGATTCTACTTT R:TGTCTTGTGACCAAATTGAC	8	8	100	0.89
EST-SSR67	F:CCACCGTTGATTCTACTTT	9	8	88.89	0.91
	R:TGAAGTGGCGGCGGAAGA				
EST-SSR69	F:ATCCACCGTATGATGCT	8	7	87.5	0.76
	R:AAGACCCATACAAAAGATACT				
EST-SSR72	F:ATCCACCGTATGATGCT	9	9	100	0.83
	R:GTCAAGTCAAAAACGCCG				
EST-SSR74	F:ATCCACCGTATGATGCT	9	8	88.89	0.88
	R:TTGGTAGCCTCTTCTTTTG				
EST-SSR76	F:GAATCAGTGAATAAAGCGTGTA	6	6	100	0.98
	R:GATGGGCTTGTCTTCGTC				
EST-SSR77	F:GAATCAGTGAATAAAGCGTGTA	7	6	85.72	0.94
	R:GCAGGTTAGCGGTGGTTA				
EST-SSR85	F:GAATCAGTGAATAAAGCGTGTA	6	6	100	0.94
	R:GATGACGATGGAGTGGG				
EST-SSR88	F:GAAAGTGCGAAACCAAAC	8	7	87.5	0.97
	R:AAGACCCATACAAAAGATACT				
EST-SSR91	F:GAAAGTGCGAAACCAAAC	5	5	100	0.95
	R:TGTCTTGTGACCAAATTGAC				
EST-SSR99	F:CAAGCAATACATACACACA	4	4	100	0.94
	R:TTGGTAGCCTCTTCTTTTG				
EST-SSR40	F:ATCCACCGTATGATGCT	5	5	100	0.98
	R:TGTCTTGTGACCAAATTGAC

**Table 2:** Comparison of the efficiency of EST-SSR, SRAP, and SCoT primers

Type of marker	SCoT	SRAP	EST-SST
Number of primers	38	40	40
Total number of bands(TNB)	414	338	320
Average number of loci per assay	10.89	8.45	8.00
Number of polymorphic bands(NPB)	398	302	301
Percentage of polymorphic bands (PPB)	96.14%	89.35%	94.06%
Polymorphism information content (PIC)	0.79	0.77	0.85
Resolving power (Rp)	6.71	5.11	4.48
Effective multiplex ratio (EMR)	10.47	7.55	7.53
Average band informativeness (Ib_av_)	0.58	0.61	0.56
Marker index (MI)	6.07	4.61	4.22

### Correlation analysis among genetic distance matrices by three-types of marker dataset

**Fig. 1:**
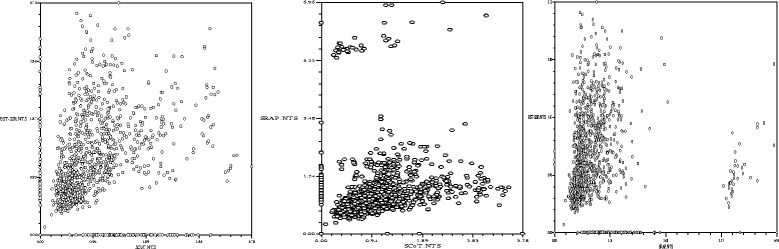
The correlation between the genetic distance matrices using Mantel tests

Mantel tests [21] were used to measure the correlation between the genetic distance matrices generated by SCoT, SRAP and EST-SSR molecular markers. *r* ≥ 0.9, 0.8 ≤ *r* < 0.9, 0.7 ≤ *r* < 0.8, and *r* < 0.7 represented significant correlation, moderate correlation, weak correlation, and no correlation, respectively. In the present study, the coefficients of correlation (r) between the genetic distance matrices of SCoT and EST-SSR markers, SCoT and SRAP markers, and SRAP and EST-SSR markers were 0.19, 0.17, and 0.01, respectively (Fig. 1). Different molecular markers and different primers of the same marker all yielded distinct amplification products, which reflected the polymorphism of the genomic regions; hence, utilization of different marker designing strategies will produce different results. Theoretically, the validity of the results should improve with increasing numbers of markers and increasing coverage of the genome. Therefore, we employed three types of molecular markers to generated 1072 bands and to perform genetic constitution analyses.

### Genetic constitution analysis

#### Analysis using STRUCTURE

**Fig. 2:**
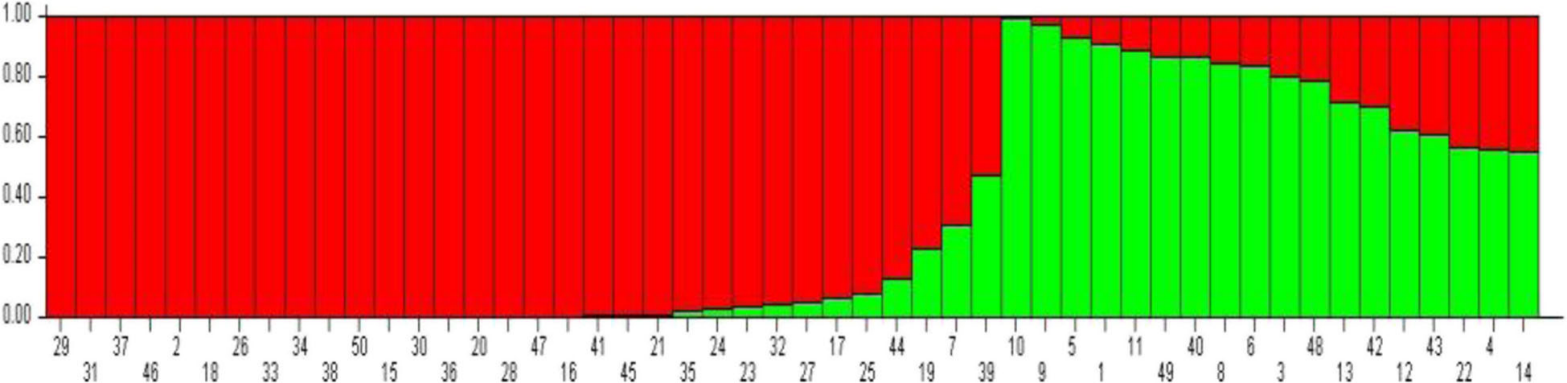
STRUCTURE analysis of the number of population for K. The number of subpopulations(k) was identified based on maximum likelihood and k values. The most likely value of k identified by STRUCTURE was observed at k = 2. Note: Green bands: Group 1, Red bands: Group 2. The proportion of each color reflects the probability that each of the test materials (numbered from 1 to 50) belongs the corresponding group

One thousand seventy-two polymorphic bands with MAF (minor allele frequency) < 5% were used to elucidate the population structure of the entire pool of tea germplasms. In this study, STRUCTURE 2.3.4, which applies a Bayesian clustering algorithm, was used to simulate population genetic structure based on the assumption that the 1072 loci were independent. Using a membership probability threshold of 0.60, population K values from 1 to 10 were simulated with 20 iterations for each K using 10,000 burn-in periods followed by 10,000 Markov Chain Monte Carlo iterations in order to obtain an estimate of the most probable number of population. Delta K was plotted against K values; the best number of clusters was determined following the method proposed by Evanno et al. [22] and obtained via the Structure Harvester platform (http://taylor0.biology.ucla.edu/structureHarvester/). Delta K reached a maximum value at K = 2, suggesting that the 50 tea germplasm were best divided into two subgroups (Fig. 2).

#### UPGMA clustering

**Fig. 3:**
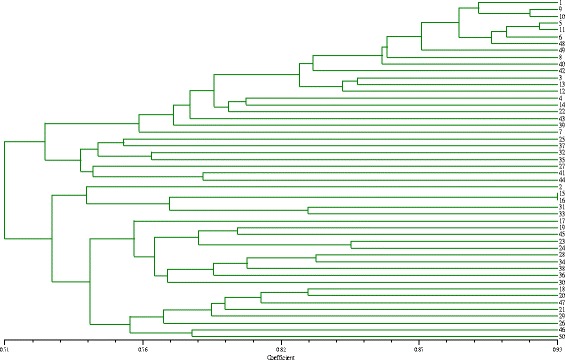
Cluster dendrogram of 50 tea genotypes constructed based on UPGMA by EST-SSR, SRAP and SCoT

A dendrogram was constructed with cluster analysis using the unweighted pair-group method with arithmetic means (UPGMA), which demonstrated that the 50 genotypes could be clearly divided into 2 groups (Fig. 3). Group I included 27 varieties, and group II contained 23 varieties. The average similarity coefficient was 0.74. The two most closely related materials were 15 and 16, which have a sister line with a genetic similarity coefficient of 0.93.

#### Principal components analysis

**Fig. 4:**
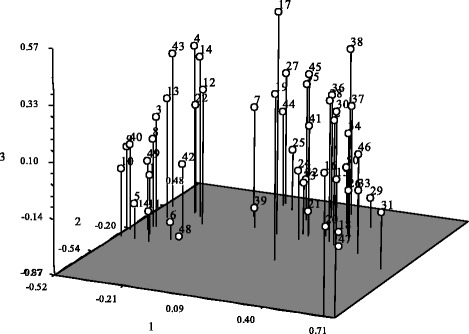
PCA plots based on the first three components

The top three principal components were used to analyze population structure. Principal component analysis was conducted under NTSYS-pc2.10e [23]. The results showed that the three PCs had contribution rates of 15.97%, 8.50% and 6.17%. PCA separated the 50 genotypes into two major groups (Fig. 4) which were consistent with the STRUCTURE and UPGMA results. GroupI consisted of 18 genotypes (Fig. 4, left), with the other 32 genotypes belonging to group II (Fig. 4, right).

The analysis performed using STRUCTURE, UPGMA and PCA yielded similar results, clustering the 50 genotypes into 2 sub-populations. Of note, PCA results had good consistency with previous results from STRUCTURE. The results generated using UPGMA were slightly different from those using STRUCTURE and PCA (Table 3) and bold numbers in group 1 by UPGMA represent the differences between the results using STRUCTURE and PCA and the results using NJ.

**Table 3:** Comparison of the clustering by STRUCTURE, PCA and UPGME

Clustering method	Code of group1	Code of group2
STRUCTURE	1,3,4,5,6,8,9,10,11,12,13,14,22,40,42,43,48,49	2,7,15,16,17,18,19,20,21,23,24,25,26,27,28,29,30,31,32,33,34,35,36,37,38,39,41,44,45,46,47,50
PCA	1,3,4,5,6,8,9,10,11,12,13,14,22,40,42,43,48,49	2,7,15,16,17,18,19,20,21,23,24,25,26,27,28,29,30,31,32,33,34,35,36,37,38,39,41,44,45,46,47,50
UPGMA	1,3,4,5,6,**7**,8,9,10,11,12,13,14,22,**25**,**27**,**32**,**35**,**37**, **39**,40,**41**, 42,43, **44**,48,49	2,15,16,17,18,19,20,21,23,24,26,28,29,30,31,33,34,36,38,45,46,47,50

Note: Bold numbers in group 1 by UPGMA method refer to the code that different from STRUCTURE and PC method

## Conclusions

We firstly reported the use of SCoT markers to analysis genetic diversity of tea germplasms. The results showed that SCoT markers revealed high genetic diversity among tea resources. In the future, we planed to select core SCoT markers. Different kinds of molecular markers can reveal different and complementary information of the same genome. Thus, we highly recommend using more marker types for comprehensive evaluation of genetic diversity and structure. 50 accessions were clustered into 2 sub-populations based on STRUCTURE, UPGMA and PCA; there was no obvious differences between imported and local germplasms. The genes of exotic varieties have been constantly integrated into the gene pool of Qinba tea through long-term (20–25 years) tea breeding and production activities. The selection of varieties with economic characters was emphasized during the process of breeding, resulting in the loss of some tea resources and the decrease of genetic diversity; thus, it is necessary to introduce new tea tree resources in order to broaden the genetic diversity.

## Methods

### Plant materials

A total of 50 tea plant genotypes, representing most tea germplasm of the Qinba area in China, were collected from the tea experimental farm of the Hanzhong Institute of Agricultural Sciences during the 2016 growing season (Table 4).

**Table 4:** The 50 tea plant samples used for marker (EST-SSR, SRAP and SCoT) genotyping

Code	Name	Origin of tea district	Code	Name	Origin of tea district	Code	Name	Origin of tea district
1	BeiBa13–2	Jiangbei	18	FuDingDaBai	Huanan	35	ZhenBa14–5	Jiangbei
2	BeiBa13–7	Jiangbei	19	YunKang10	Xinan	36	Xi14–37	Jiangbei
3	Xi13–16	Jiangbei	20	FuXiang3	Huanan	37	Xi14–36	Jiangbei
4	Ning13–6	Jiangbei	21	XiangBoLu	Jiangnan	38	ZhenBa14–19	Jiangbei
5	BeiBa12–1	Jiangbei	22	FuXuan9	Huanan	39	DaJiaoBan1	Jiangbei
6	BeiBa12–3	Jiangbei	23	ZhuYeQi	Jiangnan	40	WuNiuZao	Jiangnan
7	BeiBa11–2	Jiangbei	24	LongJing	Jiangnan	41	CuiFeng	Jiangnan
8	YuSun2	Jiangnan	25	BeiBa14–32	Jiangbei	42	BeiBa12–4	Jiangbei
9	BeiBa11–6	Jiangbei	26	BeiBa14–37	Jiangbei	43	HuangJinCha1	Jiangnan
10	ZaoBaiJian	Xinan	27	Ning14–14-2	Jiangbei	44	BaiHaoZao	Jiangnan
11	JinGuanYin	Huanan	28	Ning14–51	Jiangbei	45	JinMuDan	Huanan
12	ShanCha1	Jiangbei	29	Xi14–10	Jiangbei	46	ChunBoLu	Huanan
13	PingYangTeZao	Jiangnan	30	ZhenBa14–39	Jiangbei	47	Echa1	Jiangnan
14	DanGui	Huanan	31	ZhenBa14–22	Jiangbei	48	ChunYu1	Jiangnan
15	Ning13–3	Jiangbei	32	Ning14–50	Jiangbei	49	ShiFuCui	Jiangbei
16	Xi13–10-1	Jiangbei	33	Xi14–1	Jiangbei	50	BeiBa14–42	Jiangbei
17	Ning13–14	Jiangbei	34	Xi14–50	Jiangbei			

### DNA extraction and marker genotyping

Genomic DNA was extracted from fresh leaves of each individual using the modified CTAB technique and detected with 0.8% agarose gel electrophoresis. PCR was carried out as follows: 2 × Taq Master Mix (7.5 μL), forward and reverse primers (1 μL each, 2 μL for SCoT primers), RNase-free water (3.5 μL), and tea genomic DNA (2 μL). In order to improve the effect of PCR amplification, changing annealing temperature was used in a PCR reaction system; the reactions were programed as follows: initial denaturation at 94.0 °C for 5 min, denaturation at 94.0 °C for 1 min, annealing at 60.0 °C for 1 min, and extension at 72.0 °C for 1 min, for a total of 10 cycles; subsequently, a total of 35 cycles of denaturation at 94.0 °C for 30 s, annealing at 35 °C for 30 s, and extension at 72.0 °C for 1 min were performed. The duration of extension was 10 min; then storage at 4.0 °C. The selected primers were synthesized by Shanghai Sangon Biological Engineering Technology and Service Company (Shanghai, China). Initially, six germplasms (LongJing, ShanCha1, ChunBoLu, BeiBa11–6, Ning13–6, ZaoBaiJian) were used to screen markers for high polymorphim. Then, 40 pairs of clear and highly polymorphic EST-SSR and SRAP markers, and 38 paris of SCoT marker primers were selected from 154 EST-SSR pairs, 154 SRAP pairs, 125 SCoT pairs. Electrophoresis was performed using 8% non-denaturing polyacrylamide gel under 160 V voltage; the bands were visualized via silver staining.

### Genetic variation and marker efficiency analysis

Following electrophoresis, each amplification band corresponded to a primer hybridization locus and was considered as an effective molecular marker. Each polymorphic band detected by a same given primer represented an allelic mutation. In order to generate molecular data matrices, clear bands for each fragment were scored in every accession for each primer pair and recorded as 1 (presence of a fragment), 0 (absence of a fragment), and 9 (complete absence of band). Excel was used to compute the marker index (MI) of the three types of markers and the marker frequencies of the three types of markers were compared. MI values were obtained from the average band informativeness (Ib_av_) and the effectiveness multiplex ratio (EMR); EMR represents the number of polymorphic loci and Ib_av_ is given by the following formula:

$$ {Ib}_{av}=\frac{1}{n}\sum \limits_{i=1}^n\left(1-\left(2\left|0.5-{P}_i\right|\right)\right), $$

where P_i_ represents the proportion of the i^th^ sample in the amplified locus and n represents the total number of amplified loci. Using the method reported by Smith et al. [24], the value of the polymorphism information content (PIC) was calculated with the formula:

$$ PIC=1-\sum \limits_{i=1}^n{P_i}^2-\sum \limits_{i=1}^{n-1}\sum \limits_{j=i+1}^n2{P_i}^2{P_j}^2, $$

where PIC represents the PIC value of the i^th^ locus and P_ij_ represents the frequency that allele j appears in the i^th^ locus. The value of PIC varies from 0 to 1, with 0 indicating an absence of polymorphism at a given locus and 1 reflecting multiple alleles at a given locus. The level of polymorphism of each marker was assessed by the polymorphism information content (Botstein et al. [25]), which measures the extent of genetic variation: PIC values smaller than 0.25 indicates low levels of polymorphism associated to a locus, PIC values between 0.25 and 0.5 imply moderate levels of polymorphism, while PIC values greater than 0.5 indicate high levels of polymorphism.

### Correlation analysis among genetic distance matrices by three-types of marker dataset

Mantel test was carried out with the batch file of the NTSYS-pc2.10e software.

#### Genetic constitution analysis

STRUCTURE v2.3.4 was used to assess the population structure of the 50 tea genotypes with 1072 loci. The number of sub-population (K) was set from 1 to 10 based on admixture models and correlated band frequencies. Genetic similarity coefficients were computed using the SM functionality of the NTSYS-pc2.10e software, cluster analysis were conducted using the UPGMA method, and the principal component analysis using the batch file under the NTSYS-pc2.10e software.

## Abbreviations

EMR: Effective multiplex ratio
EST-SSR: Expressed sequence tags-Simple sequence repeats
Ibav: Average band informativeness
MAF: Minor allele frequency
MI: Marker index
NJ: Neighbor-joining
PCA: Principal component analyses
PIC: Polymorphism information content
PPB: Percentage of polymorphic bands
Rp: Resolving power
SCoT: Start codon targeted polymorphism
SRAP: Sequence-related amplified polymorphism
UPGMA: Unweighted pair group method with arithmetic mean

## References

[1] Yao MZ, Chen L, Ma CL (2009). Comparative analysis of genetic diversity among tea cultivars from China, Japan and Kenya by ISSR and EST-SSR. Mol Plant Breed.

[2] Yao MZ, Qiao TT, Ma CL (2010). The association analysis of phenotypic traits with EST-SSR markers in tea plants. J Tea Sci.

[3] Liu B, Sun X, Li Y (2009). Analysis of genetic diversity of tea plants by using EST-SSR and ISSR markers. Chin J Trop Crop.

[4] Qiao TT, Ma CL, Zhou YH (2010). EST-SSR genetic diversity and population structure of tea landraces and developed cultivars (lines) in Zhejiang Province, China. Acta Agron Sin.

[5] Li SJ, Wang X, Duan JH, Dong LJ, Zhang SG (2011). Genetic diversity and genetic structure of 16 tea cultivars based on SSR markers. Hunan Agric Sci.

[6] Li G, Quiros CF (2001). Sequence-related amplified polymorphism (SRAP), a new marker system based on a simple PCR reaction: its application to mapping and gene tagging in Brassica. Theor Appl Genet.

[7] Shen CW, Ning ZX, Huang JA (2009). Genetic diversity of Camellia sinensis germplasm in Guangdong Province based on morphological parameters and SRAP markers. Chin J Appl Ecol.

[8] Shen CW, Huang JA, Zhao SH, Ning ZX, Li JX, Zhao CY, Chen D (2010). Analysis of genetic diversity of camellia sinensis germplasm in Guangdong Province by srap and issr markers. J Nucl Agric Sci.

[9] Xi CY, Tang Q (2013). Wu YS, Xu JY, Chen H, Wu Q. Genetic diversity and relationship of 30 tea plant germplasms in Sichuan revealed by SRAP marker. Guizhou Agric Sci.

[10] Liu Z, Zhao Y, Yang PD, Chen Y, Ning J, Yang Y (2014). Comparison of parents identification for tea variety based on SSR, SRAP and ISSR markers. J Tea Sci.

[11] Chen XJ, Zhou KH, Zong HX, Fang R (2012). Genetic diversity of capsicum frutescens in China as revealed by SRAP and SSR markers. Acta Bot Bor-Occid Sin.

[12] Xia FG, Zhong XW, Wu F (2017). SRAP marker analysis of genetic diversity and relationship in Wuyi rock tea Germplasm resources. J Tea Sci.

[13] Bertrand C, David J, Mackill CY (2009). Start codon targeted (SCoT) polymorphism:a simple, novel DNA marker technique for generating gene-targeted markers in plants. Plant Mol Biol Rep.

[14] Luo T, Yang HX, Cen HF, Liu XH, Gao YJ, Duan WX (2013). Application of SCoT molecular marker in construction of molecular genetic linkage map of saccharum spontaneum L. J Plant Genet Resour.

[15] Jiang LF, Zhang XQ, Huang LK, Ma X, Yan DF, Hu Q (2014). Analysis of genetic diversity in a cocksfoot (Dactylis glomerata) variety using SCoT markers. Acta Pratacult Sin.

[16] Luo C. Study on SCoT marker and analysis on genes of stress-related and important flowering time in mango. Nanning Guangxi: Guangxi University; 2012.

[17] Xiong FQ, Jiang J, Zhong RC, Han ZQ, He LQ, Li Z, Zhuang WJ, Tang RH (2010). Application of SCoT molecular marker in genus arachis. Acta Agron Sin.

[18] Chen S. Study on genetic diversity and smut resistance evaluation of sugarcane parents. Guangzhou: South China Agricultural University; 2016.

[19] Wei YL, He XH, Luo C, Chen H (2012). Genetic diversity of podocarpus by SCoT markers. Guihaia.

[20] Hou XG, Wang J, Jia T, Zhang YQ, Hou J, Li JJ (2011). Orthogonal optimization of SCoT-PCR system and primer screening of tree peony. Acta Agriculturae Boreali-Sinica.

[21] Mantel N (1967). The detection of disease clustering and a generalized regression approach. Cancer Res.

[22] Evanno G, Regnaut S, Goudet J (2005). Detecting the number of clusters of individuals using the software STRUCTURE: a simulation study. Mol Ecol.

[23] Rohlf FJ (1998). NTSYS-pc - numerical taxonomy and multivariate analysis System.

[24] Smith S, Helentjaris T, Paterson AH (1996). DNA fingerprinting and plant variety protection. Genome mapping in plants.

[25] Botstein D, White RL, Skolnick M (1980). Construction of a genetic linkage map in man using restriction fragment length polymorphisms. Am J Hum Genet.

